# Can serum amyloid a level be used to support the Familial Mediterranean Fever diagnosis?

**DOI:** 10.1186/1546-0096-12-S1-P245

**Published:** 2014-09-17

**Authors:** Kubra Ozturk, Zelal Ekinci

**Affiliations:** 1 Department of Pediatrics Rheumatology , Kocaeli Univertsity Faculty of Medicine, Kocaeli, Turkey

## Introduction

Familial Mediterranean Fever (FMF) is a periodic fever syndrome characterized by recurrent episodes of fever and serosal inflammation .The diagnosis is still based on clinical criteria. No laboratory test is diagnostic of FMF. During the attacks increased C reactive protein (CRP), serum amyloid A (SAA), fibrinogen, beta 2 microglobulin, erythrocyte sedimentation rate (ESR) and leukocytosis is observed. The increased levels of SAA in patients with FMF during the attack free period have been reported as a sign of subclinical inflammation. Also, studies have shown that during the attack the sensitivity of SAA and CRP was similar.

## Objectives

The aim of this study was to evaluate the sensitivity of SAA and other acute phase reactants in the diagnosis of FMF.

## Methods

We reviewed the medical files of 100 patients with FMF followed up in our center in which SAA was measured; mutation analysis was performed and yet untreated. The diagnosis of FMF was established according to Livneh and Yalçınkaya criteria. Patients were divided according to the presence or absence of attack while the SAA measurement was performed. The level of white blood cells (WBC), ESR, CRP, fibrinogen and platelets that measured simultaneously with SAA were recorded. For each parameter the level above the normal range accepted as increased.

## Results

Thirty one patients were evaluated during the FMF attack and 69 patients were evaluated during the attack free period. The median levels (minimum, maximum) during the attack: SAA 178 (5 - 1720) mg/L, CRP 4.5 (1 – 15.9) mg/dl, ESR 29 (4 – 88) mm/hour, fibrinogen 4.5 (2.2 – 8.1) g/L, WBC 9100 (3910 - 26700) /mm^3^ platelets 343000 (146000 - 694000) /mm^3^ ; during the attack free period: SAA 20 (1 - 351) mg/L, CRP 0.2 (0.1 – 7.6) mg/dl, ESR 7 (1 – 48) mm/hour, fibrinogen 3.3 (1.6 – 6.5) g/L, WBC 7480 (3730 - 20000) /mm^3^ platelets 325000 (173000 - 528000) /mm^3^ . The sensitivity, specificity, positive predictive value (PPV) and negative predictive value (NPV) were calculated for all parameters. (Table 1)

**Table 1:** Sensitivity, specificity, positive and negative predictive values of acute phase reactans for predicting FMF attack

	Sensitivity	Specificity	PPV	NPV
SAA	96%	28%	37%	95%

ESR	64%	88%	71%	84%

CRP	83%	85%	72%	92%

Fibrinogen	58%	82%	60%	81%

WBC	45%	81%	51%	76%

Platelets	25%	88%	50%	72%

Table 1 Sensitivity, specificity, positive and negative predictive values of acute phase reactans for predicting FMF attack.

## Conclusion

Decreased specificity and PPV of SAA in predicting FMF attack gave the impression that SAA levels during the attack free period in FMF patient is increased. In this respect it is concluded that SAA can be used as an independent laboratory parameter to support FMF diagnosis.

## Disclosure of interest

None declared.

